# Impact of a three-week full leg cast immobilization on infant crawling kinetics and spatiotemporal parameters

**DOI:** 10.1371/journal.pone.0318106

**Published:** 2025-03-12

**Authors:** Mark D. Geil

**Affiliations:** 1 Wellstar College of Health and Human Services, Kennesaw State University, Kennesaw, Georgia, United States of America; 2 Children’s Healthcare of Atlanta, Atlanta, Georgia, United States of America; Arizona State University, UNITED STATES OF AMERICA

## Abstract

Despite its importance in the development of mobility in infants, there is a general lack of quantified data on infant crawling, and a specific lack of understanding of crawling kinetics, which includes the relative weightbearing and force generation among the four limbs. Moreover, because of the difficulty of measurement and study design, there is no longitudinal quantification of the impact of a perturbation to typical development, such as limb immobilization. This study measured kinetic and spatiotemporal outcomes in a typically developing infant prior to a fracture that necessitated a long-leg cast that immobilized the left knee and ankle, while crawling with the cast, and again one day after cast removal, and two weeks post. The study utilized a pressure-sensing mat to generate outcomes. Crawling in the cast resulted in a 37% decrease in speed, a shift of weight toward the arms (toward the right arm in particular), and a shift from double and quadruple limb support toward triple limb support. Upon removal of the cast, the unweighting and limb support patterns persisted, while speed recovered and actually exceeded baseline. Triple limb support was 12% at baseline, 28% in the cast, and 32% immediately following cast removal. Two weeks later, the value had dropped back to 17%, while speed continued to increase following a linear trend vs. age. These data provide insight into an infant’s ability to reorganize crawling kinematics, and the persistence of that reorganization following remobilization.

## Introduction

Crawling is an important part of mobility development in infants. Although multiple types of crawling have been described, the most commonly observed pattern is “hands-and-knees” crawling, which is a “four-beat gait” [1] that follows a general cycle of progression or “step” by a reference hand, followed closely in time by the contralateral knee, then the contralateral hand, and finally the ipsilateral knee (Fig 2b). Development through various modes of human infant crawling has been a focus within theoretical frameworks, such as Dynamic Systems Theory, that seek to understand how and why infants learn to move. New motor skills emerge from a child’s ability to interact with and organize multiple complex subsystems that are embedded within the individual and are open to complex conditions of the environment and the demands of the task [2,3]. As infants learn to move, these interactions become motor patterns for crawling and walking. Sporns and Edelman [4] and Edelman [5] have focused on neural connections that change as a child develops favorable patterns that are successful in the environment as a function of growth and maturation, current context, and past experiences. Initially, the child has many disorganized neuronal connections, with a high degree of variability and instability. As favorable patterns are chosen and practiced, the variability of neuronal paths diminishes and selected neuronal pathways are repeated. From development and information gained through movement in the environment, neuronal networks are chosen, grouped, and repeated, generating new goal-directed connections [4]. Adolph and colleagues studied the mobility adaptations of early infant crawlers and toddler walkers to changes in their environment [6,7]. When comparing the two groups, the crawlers did not change their motor strategy to adjust to different inclines while the walkers did. Adolph further discussed the importance of crawling, suggesting that crawling is a precursor to walking through learning by practice [6]. Crawling was also observed as a sequencing of locomotion so infants can learn the adaptations used later in walking. Adolph has also suggested that growth creates natural changes in body dimensions that make the infant’s body more stable (e.g., the center of mass is lowered relative to overall height as the body grows) and that because infants learn to crawl and walk while their body dimensions are changing, locomotion requires continual adaptation to cope with changing body size, environment, ground surfaces, and activity [8,9].

**Fig 1:**
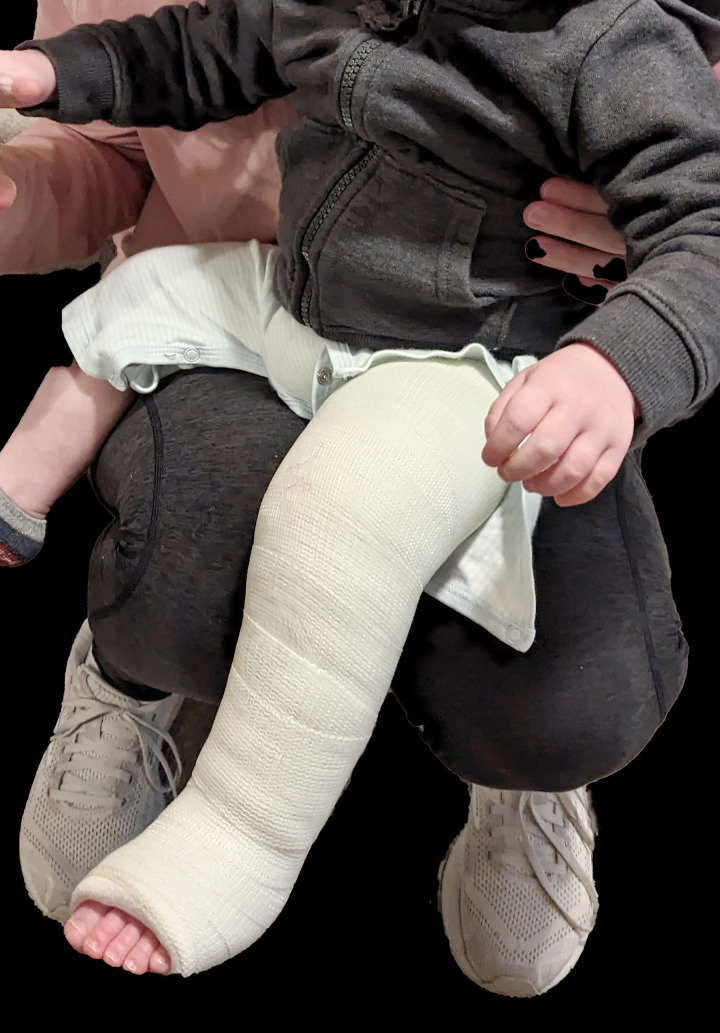
Photograph of Immobilized Limb. Photograph of subject in left semi-rigid long-leg soft cast.

A limitation of these landmark studies has been the lack of quantitative data to describe crawling development. Researchers have theorized about the role of body weight and ground reaction force on crawling development but have not actually measured it. Others have observed patterns of limb pairings [10] but without the tools needed for independent quantification. Our research group and others have assessed instrumented crawling kinematics in children with limb loss by attaching reflective markers and using motion capture [11,12]. However, we are aware of only a single study that has quantified typical kinetics (ground reaction forces) of overground crawling, a cross-sectional study of eight children in a single crawling pattern using a sensor designed for industrial purposes [13]. Patrick et al. measured limb coordination via video recording along with surface EMG and joint angles via electrogoniometers as part of a perturbation paradigm in treadmill crawling, but did not assess longitudinal development [14]. It is difficult to determine if differentiation in an infant’s crawling is significant and meaningful if comparisons can only be made using subjective observations and not objective numbers.

The purpose of this longitudinal single-case study was to understand changes in kinetic and spatiotemporal crawling parameters when an infant has one leg immobilized. Quantifying differences in a broad number of biomechanical outcomes can contribute to determining their minimal clinically important differences, and understanding the infant’s dynamic reorganization of motor patterns can provide insight into motor development not available without such quantification.

## Materials and methods

A single typically developing subject was enrolled in a larger study on the development of infant crawling. Written parental permission was obtained for participation in the larger study, which was approved by the Kennesaw State University Institutional Review Board. The individuals pictured in Fig 1 have provided written informed consent (as outlined in PLOS consent form) to publish this image alongside the manuscript. While enrolled, the subject sustained a fall at home resulting in a left distal tibia/fibula buckle fracture. The fracture was treated with a long-leg soft cast, immobilizing the knee and ankle for three weeks. The semi-rigid fiberglass cast extended from the proximal femoral area to a level just distal to the metatarsal heads (Fig 1).

The child was cleared for weightbearing with no mobility restrictions while in the cast. He returned to active crawling one day following casting. Given frequency of data collection similar to the larger study, and the child’s active household and community crawling while casted and lack of mobility restrictions, the Institutional Review Board determined that no protocol amendment was necessary for the single-subject study. Data collection for this study occurred between January 19, 2024 and March 5, 2024.

Crawling was assessed using a 4.9m x 0.6m Zeno pressure transducer mat (ProtoKinetics, Havertown, PA). The mat incorporates a distributed array of 1 cm^2^ force sensors that output 16 levels of pressure sampling at 120 Hz. PKMAS4 software, originally designed for animal-based studies of quadrupedal gait, was used for data collection and reduction. Points of contact were manually labeled as right or left arm or leg. For the legs, any potential discrete contact with the same knee and foot was combined into a single leg contact. Crawling data had been collected ten days prior to the injury (*Pre*). These data were compared to data collected eight days following casting (*Cast*), such that the first two data collection sessions were 19 days apart. Then data were collected on the morning following cast removal (*1DPost*), and again two weeks later (*2WPost*) (Table 1).

**Table 1:** Participant Information and Timing.

Session Code	Explanation	Chronology	Age (weeks)	Body Mass (kg), %ile	Length (cm), %ile
*Pre*	Baseline session prior to fx	Day 0	55.6	9.96, 50	79, 85
*Cast*	Data collection while immobilized in cast	Day 19	58.3	10.59, 70	79.5, 85
*1DPost*	Data collection the morning following cast removal	Day 32	60.1	10.05, 50	79.5, 80
*2WPost*	Data collection two weeks following cast removal	Day 46	62.1	10.01, 48	79.7, 75

Timeline of data collection sessions and participant age, body mass, and length at each session, along with normative percentiles (%ile) for mass and length based on World Health Organization Child Growth Standards for boys 0-24 months of age [15]. Body mass is inclusive of cast during *Cast* session.

In each session, the subject was encouraged to crawl back and forth along the length of the mat approximately three to five times. A representative pass that contained at least five consecutive steady-state steps was chosen for analysis in each condition. Velocity and cadence were measured for the complete pass. Step and stride length and stride width were averaged across all steps, as were the kinetic parameters, which included integrated pressure ratios for arms vs. legs and left vs. right, and diagonal pressure ratios (left arm vs. right leg and right arm vs. left leg). Finally, the percent of each limb’s cycle when the gait was in double, triple, or quadruple support was averaged across all steps and limbs.

In all conditions except for the *Cast* condition, the subject wore only a diaper. In the *Cast* condition, the subject wore cotton pants to reduce friction and protect the mat surface from scratching.

## Results

### Subject parameters

At the baseline *Pre* data collection session before the fracture, the white male subject aged 55.6 weeks was 79 cm long with a body mass of 9.96 kg (inclusive of diaper). At the *Cast* data collection session with the limb immobilized, he was 79.5 cm long with a body mass of 10.59 kg (inclusive of diaper, pants, and the additional weight of the cast). Follow up sessions (Table 1) reflect removal of cast and typical growth.

### Immobilized crawling pattern

**Fig 2:**
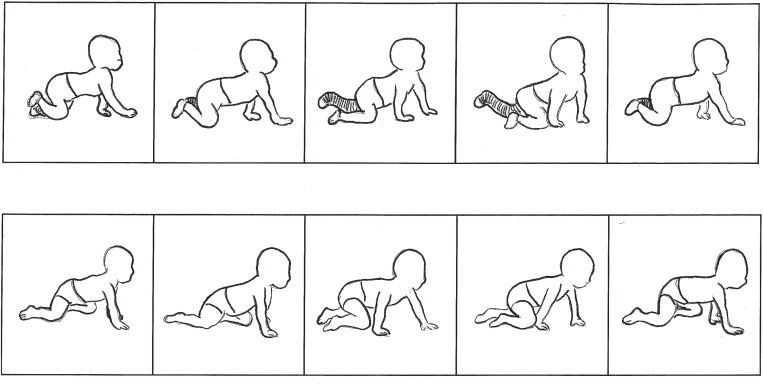
Immobilized vs. Typical Crawling Progression. Position of subject (A) and a typically developing child (B) at each point of contact in the crawling cycle, progressing through right hand contact, left knee, left hand, right knee, and subsequent right hand.

The subject was able to follow the typical four beats of the crawling cycle, except with a necessarily reduced step length for the immobilized limb due to lack of knee range of motion (Fig 2). Throughout the crawling cycle, the left (immobilized) knee never advanced past the right knee, analogous to bipedal gait with a negative step length. Despite the added weight of the cast, the subject was able to produce discrete contacts with the left leg, as opposed to dragging the limb during each swing phase.

### Spatiotemporal parameters

**Fig 3:**
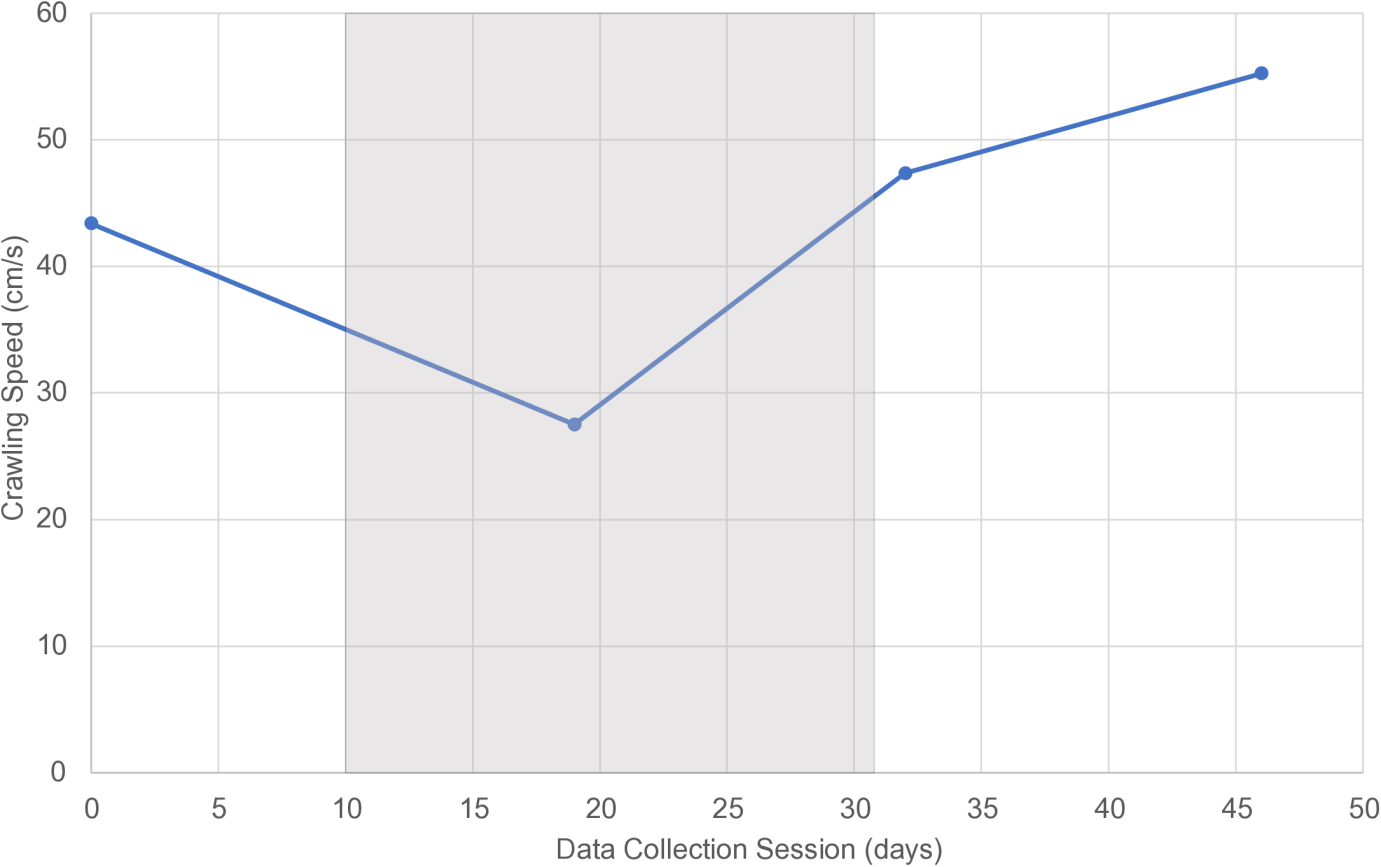
Crawling Speed. Mean self-selected crawling speed (cm/sec) at each of four time points. Gray shaded area indicates the time the limb was immobilized in the cast.

The subject crawled 37% slower than baseline in the *Cast* condition (43.4 vs. 27.5 cm/s). Cadence was similarly reduced by 31% (306.4 vs. 211.3 steps/min). Step length, the other component of crawling speed, was similar between conditions (15.69 cm for *Pre* vs. 15.49 cm for *Cast*). Crawling speed recovered upon cast removal and maintained a positive trajectory (Fig 3).

**Fig 4:**
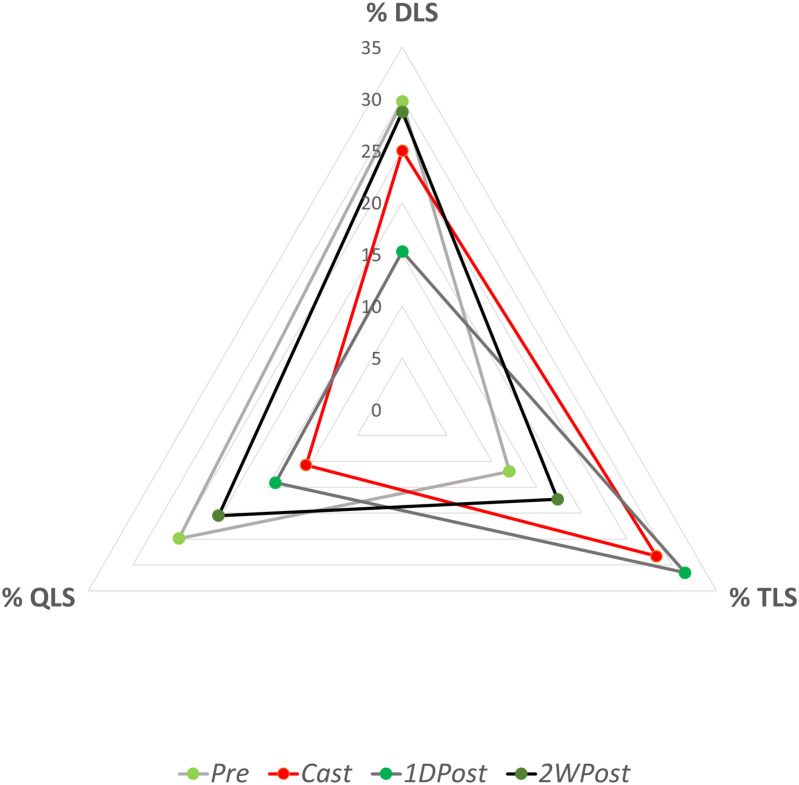
Limb Support Phases. Average for each limb and each step for percent of the gait cycle in double (% DLS), triple (% TLS), and quadruple (% QLS) support in each of the four conditions. The red line indicates the *Cast* condition, while the progressively darker gray lines with green markers indicate the conditions before and after the immobilization period.

While walking gait is divided into periods of double limb support (when both legs are in contact with the ground) and single limb support (when the contralateral limb is in swing phase) [16], crawling gait consists of periods of double, triple, and quadruple limb support. These are calculated as a percentage of the cycle for each limb and reflect the time when one, two, or all three additional limbs are in contact with the ground during the reference limb’s stance phase. At baseline, the subject’s largest support state was double-limb support, followed by quadruple limb support (Fig 4). With the cast, a large shift occurred toward triple limb support. This pattern was maintained immediately upon cast removal and recovered closer to baseline after two weeks.

### Kinetic parameters

**Fig 5:**
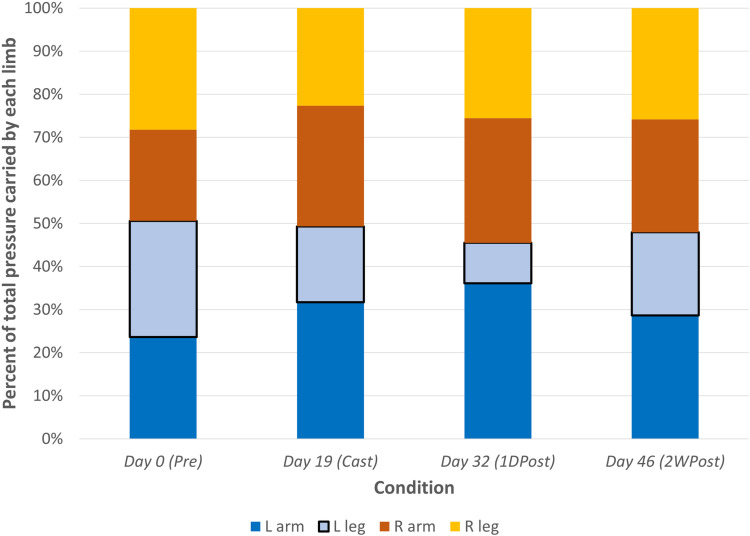
Weight Carriage. Average percent of body weight borne by each of the four limbs at each condition. The left leg (shown with a back border) was immobilized for three weeks during the *Cast* condition.

Weightbearing on the affected limb was unchanged from baseline in the *Cast* condition, despite the additional weight of the cast (Fig 5). At *1DPost*, the left leg was unloaded by 72%. At *2WPost*, weightbearing on the left leg had recovered, but still showed a 36% reduction compared to *Pre*.

Weightbearing pressure ratios also showed the effect of immobilization (Table 2). The “diagonal” pressure ratio, Right Arm/Left Leg, was most affected, with a baseline value of 0.78, a shift away from the casted leg and toward the contralateral arm during *Cast* to 1.60, a substantial increase immediately following cast removal to 3.08, and a recovery after two weeks to 1.38. The effect was also seen in fore-aft weightbearing, offloading the legs at *Cast* and *1DPost*. Bilaterally, the subject shifted slightly toward the right side, but resumed a symmetrical pattern at *2WPost*.

**Table 2:** Mean Integrated Pressure Ratios.

Ratio	*Pre*	*Cast*	*1DPost*	*2WPost*
Arms/ Legs (Fore-aft symmetry)	0.815	1.465	1.961	1.228
Left/ Right (bilateral symmetry)	1.028	0.987	0.829	0.991
Left arm/ Right leg (diagonal)	0.835	1.401	1.411	1.168
Right arm/ Left leg (diagonal)	0.784	1.595	3.083	1.377

Comparisons of the amount of body weight supported by each of the four limbs. A value of 1 indicates perfect symmetry.

## Discussion

This study was the result of a unique opportunity to capture kinetic and spatiotemporal outcomes of crawling before, during, and after a period of lower limb immobilization. Our analysis requires discrete points of contact on the pressure mat. For example, spatiotemporal parameters cannot be measured if a limb is dragged along as opposed to being lifted off the ground, engaging in a swing phase, and making contact for the next cycle. Fortunately, the subject demonstrated discrete points of contact in all conditions, enabling the analysis.

To our knowledge, this is the first instrumented longitudinal analysis of crawling measuring typical gait outcomes. While previous work has assessed infants observationally, or using measures like the Neuromotor Behavioral Inventory [17], none has assessed the implications of immobilization, making comparison of results to existing literature difficult. Yozu et al. found similar anterior/posterior weightbearing ratios as our baseline values [13], and our crawling speeds were on the high end of those found by Righetti et al., which is not unexpected because they tested a younger population [12]. Both Xiong et al. and Kawashima et al. measured walking speed versus age, but data were only available in imprecise bar and line graphs. In a single subject, Kawashima et al. reported an increase in crawling speed from approximately 0.3 to 0.5 m/s over a 0.8-month span [18]. Xiong et al. grouped infants into three age ranges (less than 9 months, between 9 and 12 months, and greater than 12 months) and reported average speeds of approximately 0.2, 0.25, and 0.4 m/s [19]. By comparison, the subject in the current study showed an overall progression in speed of 0.12 m/s over a span of approximately 1.5 months, which is less than Kawashima’s single subject, but could be considered comparable to Xiong et al.’s progressions.

The cast added approximately 0.6 kg to the child’s mass; however, the amount of body weight borne by the left leg during crawling was reduced relative to the other limbs between *Pre* and *Cast* conditions, indicating an unweighting strategy. There are at least two possible explanations. First, the child could choose to reduce load on the leg as a protection mechanism due to the fracture. Second, the load might be reduced due to the limb’s inability to accomplish the typical knee flexion used for propulsion. These modified kinetics were likely associated with the altered limb support, with a shift toward triple limb support in all three conditions following baseline. A typical infant crawling cycle involves motion of an arm and contralateral leg at the same time. If these contacts are consistently near-simultaneous, the resulting limb support profile is dominated by double limb support (when the two limbs are advancing) and quadruple limb support (the phase immediately following contact). For the child in this study, triple limb support was used to unweight the left leg, or to augment its missing propulsion while immobilized. Immediately following cast removal, the child increased mean percent triple limb support, which was unexpected. Observation revealed a general lack of left knee flexion. It can be concluded that the child maintained the kinematics learned during leg immobilization. Despite this, crawling speed returned to a value exceeding baseline. It is therefore likely that, although the left leg was not contributing substantially to propulsion, the reduced bulk and weight following cast removal enabled a carryover motion pattern to accomplish more speed.

At the *2WPost* condition, the child had returned to a typical developmental progression of increased crawling speed, cadence, and step length. In fact, considering speed at only the *Pre, 1DPost,* and *2WPost* conditions vs. age, the three data points showed a linear correlation of 1.64 cm/sec gain per week, R^2^ =  0.84.

Adults who face acute alterations in limb function such as pain or immobilization rapidly modify their gait kinematics and muscle firing patterns to adjust to the alteration. The data in the present study represent an infant crawler doing the same thing; however, the paradigm is different in infants for at least two reasons. First, limb and body mass, along with ratios of muscle to fat in infants’ legs, play a role in the development and organization of various stepping patterns [20]. The addition of a cast affects this balance. Second, infant crawling develops during the neuroplastic window for motor learning [21], In particular, crawling is a volitional movement that involves central nervous system control of rhythmic muscle contractions [1]. Therefore, understanding plastic versus elastic neural changes that result from changes in environment or limb function is important. In children with hemiplegic cerebral palsy, asymmetrical development results from both the primary deficit and from subsequent lack of use, called “developmental disregard” [22]. Although the acute and temporary constraint caused by limb immobilization is scarcely analogous to cerebral palsy, the vulnerable period of developmental neuroplasticity remains important. If new movements are learned by an infant during immobilization, sensory and motor neural pathways could reorganize, which must be followed by a new reorganization following immobilization, as opposed to an adult who is simply relearning prior patterns.

In this single case (based on observed motion and not directly measured muscle firing), muscle firing patterns lingered following immobilization. Unweighting of the limb also persisted but was resolving. This underscores the importance of therapy following significant longer-term injury or cessation of immobilization, and the need for future research focused on the effects of short- and long-duration immobilization on neuroplasticity in developing children.

The study had several limitations. Foremost, the unique circumstances meant that only a single subject was included. The results cannot therefore be generalized to a larger population. During crawling, children progress rapidly through stages of motor development, and even typically developing infants show considerable variability. Nonetheless, these data represent the first kinetic quantification of a case like this one, and the results provide insight into the mechanical effects of immobilization and the recovery time that was not available before, even for a single case.

During data collection in the *Cast* condition, the subject wore cotton pants over the cast. In all other conditions, the subject wore only a diaper. This change was made to protect the surface of the mat and to normalize friction, since the focus was on the immobilization and not the surface properties.

Finally, the study did not independently measure changes in strength or muscle atrophy in the immobilized limb, and implications on relative contribution to propulsion are speculative.

In conclusion, the subject in this study showed several modifications while crawling with an immobilized limb. Most prominently, he was slower, reduced loading of the involved limb, and shifted his temporal strategy toward greater triple limb support. Immediately following cast removal, he maintained his off-weighting strategy but regained speed. Two weeks later, his speed gains followed an almost linear progression with age, while his kinetic patterns had not quite fully recovered.
